# Buffy coat specimens remain viable as a DNA source for highly multiplexed genome-wide genetic tests after long term storage

**DOI:** 10.1186/1479-5876-9-91

**Published:** 2011-06-10

**Authors:** Josyf C Mychaleckyj, Emily A Farber, Jessica Chmielewski, Jamie Artale, Laney S Light, Donald W Bowden, Xuanlin Hou, Santica M Marcovina

**Affiliations:** 1 Center for Public Health Genomics, University of Virginia, Charlottesville, VA, USA; 2 Northwest Lipid Metabolism and Diabetes Research Laboratories, University of Washington, Seattle, WA, USA; 3 Division of Public Health Sciences, Wake Forest University School of Medicine, Winston-Salem, NC, USA; 4 Center for Diabetes Research, Wake Forest University School of Medicine, Winston-Salem, NC, USA

## Abstract

**Background:**

Blood specimen collection at an early study visit is often included in observational studies or clinical trials for analysis of secondary outcome biomarkers. A common protocol is to store buffy coat specimens for future DNA isolation and these may remain in frozen storage for many years. It is uncertain if the DNA remains suitable for modern genome wide association (GWA) genotyping.

**Methods:**

We isolated DNA from 120 Action to Control Cardiovascular Risk in Diabetes (ACCORD) clinical trial buffy coats sampling a range of storage times up to 9 years and other factors that could influence DNA yield. We performed TaqMan SNP and GWA genotyping to test whether the DNA retained integrity for high quality genetic analysis.

**Results:**

We tested two QIAGEN automated protocols for DNA isolation, preferring the Compromised Blood Protocol despite similar yields. We isolated DNA from all 120 specimens (yield range 1.1-312 ug per 8.5 ml ACD tube of whole blood) with only 3/120 samples yielding < 10 ug DNA. Age of participant at blood draw was negatively associated with yield (mean change -2.1 ug/year). DNA quality was very good based on gel electrophoresis QC, TaqMan genotyping of 6 SNPs (genotyping no-call rate 1.1% in 702 genotypes), and excellent quality GWA genotyping data (maximum per sample genotype missing rate 0.64%).

**Conclusions:**

When collected as a long term clinical trial or biobank specimen for DNA, buffy coats can be stored for up to 9 years in a -80degC frozen state and still produce high yields of DNA suitable for GWA analysis and other genetic testing.

**Trial Registration:**

The Action to Control Cardiovascular Risk in Diabetes (ACCORD) trial is registered with ClinicalTrials.gov, number NCT00000620.

## Background

Clinical trials and prospective observational cohort studies are complex to design and costly to implement, hence there is a strong desire to maximize overall clinical and scientific return on investment. A common strategy is to include blood specimen collection at a baseline or early participant study visit to enable future ancillary studies or analysis of secondary biomarker outcomes. The blood specimens may be processed to produce aliquots of sera, plasma, or blood cell pack that are stored frozen for future use. For genetics studies, DNA is more stable under long-term freezer storage, but in many existing or completed studies, the study protocol required the extraction and storage of buffy coats (aliquots of white blood cell pack) [1,2]. The Action to Control Cardiovascular Risk in Diabetes (ACCORD) clinical trial is one such study that banked buffy coat specimens for future use in genetic ancillary studies. Several studies have demonstrated a decreased DNA yield with frozen storage over time [3-5].

The ACCORD trial was a randomized, multicenter, double 2 × 2 factorial design which recruited 10,251 type 2 diabetes patients that were randomized to glycemic interventions, of which 5,518 were randomized to lipid interventions in one 2 × 2 trial and 4,733 randomized to blood pressure interventions in the second 2 × 2 trial [6,7]. The trial was designed to test the effects on major cardiovascular disease events of intensive glycemia control, treatment to increase HDL-cholesterol and lower triglycerides, and intensive blood pressure control (in the context of good glycemia and LDL control). Recruitment occurred in two phases, January - June 2001 (Vanguard Phase, N = 1,174), and February 2003-October 2005 (N = 9,077) [8]. Participants were recruited, randomized, treated, and followed through a system of seven Clinical Center Networks (CCNs). Each CCN consisted of a network of collaborating clinical sites.

The trial protocol required clinics to collect a single 8.5 ml Acid Citrate Dextrose (ACD) tube of whole blood from trial participants who consented to the use of a blood specimen for future genetics studies. The specimens were refrigerated, shipped, and processed to yield buffy coats, which were stored frozen at -80 degC. These specimens have been in storage for variable periods of time, and up to 9 years for the trial Vanguard phase participants. It was unclear whether they had degraded significantly and were no longer viable for modern highly multiplexed GWA genotyping assays that simultaneously genotype 1 million SNPs and CNV probes (or more). We designed this study to answer two specific questions:

1. What was the total yield of DNA that could be expected from the buffy coat specimens collected and stored under the ACCORD trial protocol?

2. Was the isolated DNA still of sufficient quality to provide a substrate for multiplex GWA genotyping?

We isolated the DNA from 120 ACCORD trial buffy coat specimens selected from all 8 sub-arms of the trial to sample a range of storage times and other study factors that could predict total DNA yield. We performed individual SNP genotyping on aliquots from all 120 specimens and a GWA genotyping assay on 32 of the 120 to test whether the isolated DNA has retained molecular integrity for high-quality GWA study analysis.

## Methods

### Buffy Coat Specimen Collection and Storage

One 8.5 ml ACD tube of whole blood was collected from ACCORD participants during their baseline trial visit and refrigerated at 4 degC at the recruitment clinic until shipment. Institutional Review Board approval was obtained from all recruitment, laboratory, or data management sites and written informed consent was obtained from study subjects. The shipping protocol required the clinics to ship the blood tube on cold pack refrigerant to the ACCORD Central Laboratory on the same day as collection by overnight courier (within 24 hours). All buffy coats, without exception, were extracted on the day of receipt at the Central Laboratory. Processing and storage of the buffy coat fraction (white blood cell layer) was performed following the recommendations of the NHLBI Working Group [1]. Briefly, the ACD tube was centrifuged at 2000 rpm for 30 mins and the plasma removed. Using a sterile transfer pipette, the buffy coat layer was transferred to a sterile barcoded cryovial and placed on ice. An equal volume of cell freezing solution (99% glycerol, 50 nM sodium citrate, 20 mM sodium phosphate monobasic, monohydrate, and 20 mM sodium phosphate, dibasic, anhydrous) was added and the cells suspended by gentle rocking. The cryovial containing the cells was immediately transferred to a -80 degC REVCO Ultima freezer (Thermo Fisher Scientific Inc., Waltham, MA) with audible/visual warning for power failure and temperature deviation beyond set points. The freezer was monitored by a 24 hr alarm monitoring company, with monthly testing for alarm system operation. The freezer was connected to a 100 KW on site backup generator with automatic failover in case of a power outage.

Prior to DNA isolation, 120 stored frozen buffy coat specimens were randomly selected by the ACCORD Coordinating Center out of 6,008 participants who had consented to the broadest categories of genetics study usage of their specimen. The specimens were sampled with even distribution across a range of blood sample storage duration and trial assignment. There were fewer specimens available for selection in the period 2006-2009 than from earlier years (none were drawn in 2002). An equal number of specimens were selected from each of 5 time periods (2001, 2003, 2004, 2005, and 2006-2009) to sample a range of storage durations. No more than 3 participants were selected per clinic site. The specimen characteristics are shown in Table 1. The 120 sampled individuals are representative of the overall trial pool: 35% female (39% trial); 64% white (62% trial); 19% African American (19% trial); mean age at draw 63.5 years (62.2 years trial).

**Table 1:** Clinical characteristics of the stored buffy coat samples selected for DNA isolation, N = 120 total samples

Buffy Coat Sample Characteristic	N (% or Std Err)
**Gender:**	
Male	78 (65%)
Female	42 (35%)

**Race:**	
White	77 (64.2%)
African-American	23 (19.2%)
Hispanic	7 (5.8%)
Asian	5 (4.2%)
Other	8 (6.7%)

**Age at Draw:**	
Mean years (Std Err)	63.5 (0.66)
Range years	43-80

**Year Drawn:**	
2001	24 (20%)
2003	24 (20%)
2004	24 (20%)
2005	24 (20%)
2006	11 (9.2%)
2007	10 (8.3%)
2008	3 (2.5%)

**Recruiting Clinical Center Network (CCN):**	
Total CCNs Sampled	7
Mean Samples per CCN (Range)	17.1 (9-20)

**Recruiting Clinic:**	
Total Clinics Sampled	74
Mean Samples per Clinic (Range)	1.62 (1-3)

**Laboratory Receipt Time:**	
Mean hours (Std Err)	29.3 (1.52)
Range hours	12-144

Percentage sums may differ from 100% due to rounding. The Laboratory Receipt Time is the time in hours from blood collection to receipt in the Central Laboratory. The value in the parentheses after the count value in the column labeled N is the percentage or standard error except where noted as a range.

### DNA Isolation Protocol

Frozen buffy coat specimens were shipped to University of Virginia Center for Public Health Genomics for DNA isolation and GWA genotyping. DNA was isolated using automated purification protocols on a QIAGEN^® ^Autopure LS^®^. Two initial test runs of 8 samples each were performed to compare candidate isolation protocols:

1. QIAGEN^® ^Automated purification of DNA from fresh or frozen buffy coat on the Autopure LS^® ^(protocol version AP03 Nov-07, up to 10 ml sample)

2. QIAGEN^® ^Automated purification of DNA from compromised blood samples on the Autopure LS^® ^(protocol version AP06 Nov-07, up to 10 ml sample).

(Protocol documents are available at http://www.qiagen.com). According to the vendor description, protocol 1. for fresh or frozen buffy coats is applicable for samples frozen at -80 degC directly after collection and stored for less than 2 years at this temperature. The main differences between the protocols are that the compromised blood protocol 2 dispenses additional RBC lysis reagent (40 ml versus 35 ml total volume) and incubates for 30 seconds longer during lysis; uses 4 ml protein precipitation solution versus 3.34 ml during WBC lysis/protein precipitation step and centrifuges at 3000 g for 5 min versus 2 mins; centrifuges for 5 mins versus 2 mins at 3000 g during initial DNA precipitation step; and during DNA wash, uses 12 ml 70% ethanol versus 10 ml, and centrifuges for 5 min versus 1 min at 3000 g after alcohol wash to re-precipitate the DNA. Since the results from the compromised blood protocol 2 were superior, the compromised blood protocol was used for the remaining 104 samples. The remaining samples were processed in runs of 16 samples at a time.

### DNA Quality Control

After isolation and purification, the DNA was quantitated on a NanoDrop 8000, to measure concentration and assess the purity of the DNA through standard A260/A280 and A260/A230 ratios. The DNA was diluted to 400 ul total, except where yields were lower. A 3 ul aliquot of the DNA solution was evaluated for DNA length distribution and potential degradation by electrophoresis on a 1% agarose gel against a molecular weight ladder with ethidium bromide staining.

### SNP Genotyping QC

One hundred and seventeen DNA specimens were tested for success in genotyping individual SNPs using Applied Biosystems TaqMan^® ^assays. Three (3/120) samples were not genotyped due to low total DNA yield (1.07 ug, 4.00 ug, 5.22 ug) and the need to preserve the DNA for future disease genetics studies. The TaqMan genotyping assay is a QC test of the suitability of the isolated DNA for single SNP genotyping. Failure in this step indicates that the DNA quality is unlikely to be sufficient for highly multiplexed GWA genotyping. A limited panel of 6 high heterozygosity autosomal SNPs located on different human chromosomes was selected for this purpose. Applied Biosystems TaqMan^® ^Genotyping Assay Protocol (Part Number 4332856 Rev. C 05/2006) was used to genotype the SNPs on an Applied Biosystems 7900HT Fast Real-Time PCR System using standard reagents and standard cycling protocols. The SNPs are listed in Table 2.

**Table 2:** SNP panel composition and genotyping results

dbSNP rs number)	ABI TaqMan Assay Identifier	Genome Location	Samples Genotyped	Missing Genotypes (%)
rs7792547	c_29193799_10	Chr7: 150754462	117	4 (3.4%)

rs7326634	c_306605_10	Chr13: 85638600	117	1 (0.85%)

rs1293288	C_8339785_10	Chr8: 11755937	117	0 (0%)

rs6935566	c_29104855_10	Chr6: 149608518	117	1 (0.85%)

rs2251110	c_8793799_20	Chr15: 31385829	117	0 (0%)

rs2118922	c_15975275_10	Chr4: 178404068	117	2 (1.7%)

The table lists the dbSNP rs number, ABI identifier, genome location, number of samples assayed, and the missing genotype count and proportion for the 6 SNPs assayed by TaqMan genotyping. (ABI or Applied Biosystems Inc, is now part of Life Technologies).

### GWA Genotyping Assay

Thirty two specimens were randomly pre-selected by the ACCORD Coordinating Center for GWA genotyping before DNA isolation results were available. After DNA isolation 5 of these were found to have total DNA yield < 50 ug. To conserve these for future analysis, we substituted 5 higher yield specimens (yield > 50 ug) that matched the substituted specimen characteristics as far as possible with respect to year, gender, race (4/5 matches), and recruiting CCN. The 32 samples were genotyped on 8 Illumina Human Omni1-Quad beadchips, each beadchip assaying four samples for 1,140,419 SNPs and CNV probes. A minimum of 200 ng of DNA is required per sample http://www.illumina.com/documents/products/datasheets/datasheet_humanomni1_quad.pdf. The genotyping assay was performed according to the standard Illumina Infinium HD Super Assay protocol (Infinium HD Super Assay Protocol Guide, Catalog #WG-901-4002 Part# 11322427 Rev.B).

### GWA Genotyping Assay QC

The quality of the GWA genotyping data was assessed using the vendor built-in positive and negative quality control steps in the Illumina GenomeStudio software suite. Seven GWA genotyping assay controls included with every Illumina Infinium HD array monitor amplification, hybridization, extension, stripping, and staining which are assessed using the GenCall dashboard [9]. These were visually inspected for all sample GWA assays http://www.illumina.com/software/genomestudio_software.ilmn. The Gentrain2 algorithm was used for SNP quality scoring and the genotypes were also curated according to standard vendor genotyping QC protocol. Since only 32 samples were clustered, the standard cluster file (HumanOmni1-Quad_v1-0_B.egt) was used as per vendor recommendations for projects with less than 100 samples (Illumina Technote "Infinium Genotyping Data Analysis" http://www.illumina.com/Documents/products/technotes/technote_infinium_genotyping_data_analysis.pdf. SNP curation was performed following recommendations in the same document. This protocol identifies SNPs that should be manually reviewed by an experienced technician. All genotypes for poorly performing SNPs were set to missing. A separate cluster analysis was performed for X chromosome SNPs.

### GWA Statistical Genotype QC Analysis

After the genotyping laboratory QC was complete, data was exported from Illumina GenomeStudio for additional QC and statistical analysis. This QC mirrored the standard steps used for genotype data QC in many GWA studies to control the type 1 error rate associated with multiple testing of many thousands of SNPs [10,11]. The statistics included genotype missing rates by sample and by SNP.

## Results

### Isolated DNA Yields

We were able to isolate DNA from all 120 buffy coat specimens, with varying total yield. Since the buffy coat specimens had been in storage for range of durations up to 9 years, we tested two automated DNA purification protocols on a QIAGEN Autopure LS, 1) fresh or frozen buffy coat and 2) compromised blood sample. We compared the two protocols by comparing the yield and assay performance on a randomly selected subset of 8 samples for each protocol. We found no significant difference in the mean yield between the first subset, isolated using the buffy coat protocol, and second compromised blood protocol subset, mean yield (+/-sem) 139.3 +/- 9.0 ug and 162.5 +/- 9.8 ug respectively (Welch t-test p = 0.55); or between the first 8 and the remaining 112 isolated using the Compromised Blood Protocol, mean yield 139.3 +/-9.0 ug and 134.4 +/-0.6 ug (p = 0.86). However the Buffy Coat protocol group of 8 samples appeared to contain protein contamination after purification, did not rehydrate well, and had to be re-purified manually. The second group showed clean pellets and dissolved into solution without difficulty, hence we chose this protocol for automated purification of the rest of the samples.

**Figure 1:**
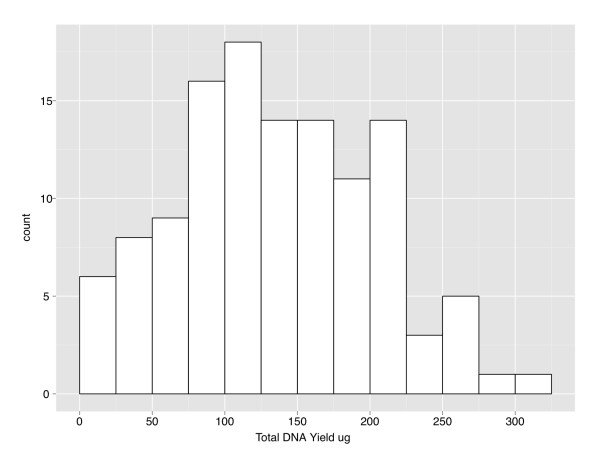
**Distribution of total DNA yield from 120 ACCORD buffy coat specimens**. The histogram shows the number of samples within each interval of total DNA yield. The interval size is 25 ug, and the maximum and minimum yields were 1.1 ug and 312.2 ug.

The distribution of total yield from all 120 samples is shown in Figure 1. For the 112 Compromised Blood Protocol specimens, the range of yields was 1.1-312.2 ug. Thirteen samples (11.6%) yielded < 50 ug, while 3 samples (2.67%) produced a yield of < 10 ug of DNA. For all 120 samples including the 8 isolated by Buffy Coat protocol, 14 yielded total DNA < 50 ug (11.7%), 4 samples yielded < 10 ug (3.3%), and 111 (92.5%) had sufficient yield to dilute into 400 ul total for future DNA stock solution (minimum required concentration 100 ng/ul). The lowest yield samples were diluted into 50 ul total stock to allow for multiple future aliquots but with variable lower concentrations. The mean yield for all 120 samples was 134.7 ug +/-0.6 ug and median was 130.6 ug.

**Figure 2:**
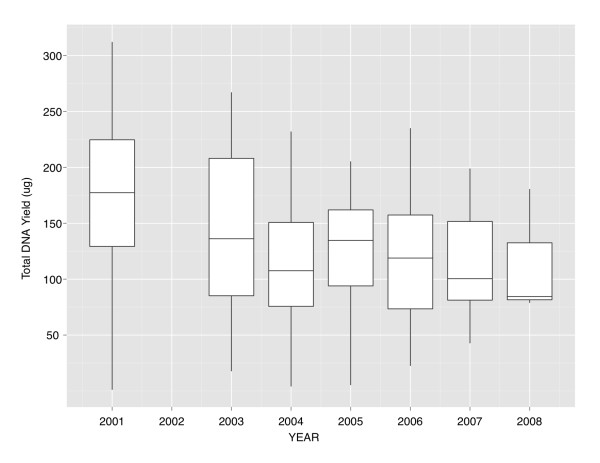
**Total DNA yield as a function of collection year from 120 ACCORD buffy coat specimens**. The figure shows a Tukey boxplot of variation in DNA yield for each year of blood collection in the 120 ACCORD samples. The upper and lower edges (hinges) of the boxes are the third and first quartiles and the central line shows the median value of yield for a year. The lines radiating above and below the boxes visually show the range to the maximum and minimum yields. There are no outlier samples in any of the years with unusually large or small yields as defined by the usual robust test of more than 1.5 × interquartile range.

To investigate the effect of study or participant factors on the yield, we tested linear regression models of total DNA yield. Figure 2 shows the yield for the different years of collection. We dropped 3 samples collected in 2008 because of insufficient cases, and recoded Asian (N = 5) and Hispanic (N = 7) samples as race "Other", ie non-White or African-American, giving White = 75, African-American = 22, Other = 20 (N = 5 Asian + 7 Hispanic + 8 coded Other in trial). Results for multivariate analysis of total DNA yield are shown in Table 3. There was no marginal association of yield with race (p = 0.16), gender (p = 0.3), clinical center network (p = 0.28), or lab receipt time (p = 0.16) after adjustment for other factors. However in the same model, year of blood collection was negatively associated with yield (beta = -11.6 ug per year +/- 4.2, p = 0.009), and age at collection was also negatively associated (beta = -2.1 ug per year +/- 0.8, p = 0.015). After dropping the 2001 specimens, year of collection was no longer significant (beta = -0.7+/- 8.3 ug, p = 0.9). Collectively these factors explain 23.5% of the total yield variance (F_12,104 _= 2.66, p = 0.004). We discuss the surprising dependency of total yield on year of collection below.

**Table 3:** Combined linear regression and analysis of variance results for predictor variables of the total DNA yield (ug)

Predictor	Number of Observations	Effect Size (Std Err)	t-test p-value	anova df	anova p-value
**Race (AfrAm)**	22			2	0.16
**White**	75	-7.4 ug (15.8)	0.21		
**Other**	20	25.7 ug (20.6)	0.64		

**Year**	117	-11.6 ug (4.2)	0.0061	1	0.0061

**CCN**	117			6	0.28

**Gender (Female)**	42				0.30
**Male**	75	13.8 ug (13.1)	0.30	1	

**Age**	117	-2.1 ug/year (0.84)	0.015	1	0.015

**Lab Receipt Time**	117	-0.7 ug/hour (0.5)	0.16	1	0.16

The table contains the marginal univariate t-test results are shown for all predictor variables with 1 or 2 degrees of freedom (df). The marginal tests describe the effect of the predictor variable after adjusting for all other variables in the model. The individual t-test results for each CCN are not shown. The anova and t-test p-values are necessarily identical for 1 degree of freedom predictors. The reference category for the categorical predictor variables (Race and Gender) are shown in parentheses beside the variable name.

### DNA Quality Control

The isolated DNA was also assessed for other factors indicative of DNA quality. The NanoDrop measured A260/A260 ratios for the 120 samples were found to be in the range 1.76-2.88, mean 1.86 and median 1.84. Three samples had ratios greater than 1.9 (2.63, 2.88, 2.05), and.all 3 of these samples also had total DNA yields < 10 ug. The results of the DNA fragmentation analysis using agarose gel electrophoresis produced distinct, bright, homogeneous bands of high molecular weight qualitatively indicating DNA that was non-degraded, free of smearing caused by presence of low molecular weight RNA, and of good quality (data not shown).

### SNP Genotyping QC

We genotyped 117 specimens in 6 SNP TaqMan assays. There were 8 total missing genotypes giving an overall missing genotype rate of 8/(117 × 6) or 1.1%. The maximum missing genotype rate per specimen was 2/6 genotypes (33%). This sample had a total yield of 109.8 ug and therefore the higher missing rate does not appear to be coincident with a low total yield. Table 4 summarizes the genotyping results for each SNP. The maximum missing rate per SNP was 3.4% (4/117) although this rate per SNP is not higher than expected for an overall per genotype missing rate of 1.1% (1-sided binomial test p = 0.01, multiple testing corrected alpha = 0.05/6 SNPs = 0.0083). We did not repeat the failed genotypes.

**Table 4:** ACCORD Illumina Omni1-Quad Statistical Genotype QC Analysis Results

Step	Samples Remaining	SNPs Remaining	Dropped (% of Remaining)
Post-Laboratory QC	32	1,140,419 (inc CNV probes)	

Remove CNV probes (not analyzed)		1,048,713	91,706

Drop SNPs with 100% missing data	32	1,015,235	33,478 (3.3%)

Drop samples with > 1% missing data	32	1,015,235	0 (0%)

**Total**	**32**	**1,015,235**	

**QC Statistic**	**N**	**Value**	**Comments**

Mean per sample Genotype Missing Rate (range; sem)	32	0.20% (0.07-0.62%; 0.024%)	For all SNPs
		0.21% (0.07-0.64%; 0.025%)	SNPs with < 100% missing genotypes

Median per sample Genotype Missing Rate	32	0.16%	

Mean per SNP Genotype Missing Rate (range; sem)	1,015,235	0.21% (0-37.5%; 0.0012%)	32 Samples

Median per SNP Genotype Missing Rate	1,015,235	0%	32 Samples

The upper part of the table shows the samples and SNPs that were dropped at each step of the Statistical Genotype QC analysis and the total number remaining. The lower part of the table summarizes values of the key QC statistics in the samples and SNPs remaining after the QC steps.

### GWA Genotyping Assay QC

The assays for all 32 samples were assessed according to the 7 genotyping assay controls included with every Illumina Infinium HD array to monitor amplification, hybridization, extension, stripping, and staining. There were no obvious poorly performing samples out of the 32 total for any of control measures. Manual curation of GWA genotype data by an experienced laboratory manager using standard Illumina GenomeStudio software and protocols identified 1,186 SNPS that passed initial automated software QC but showed overlapping or indistinct genotype cluster separation that could lead to significant errors in genotype assignment and inflated type 1 error rate in a GWA study. The genotypes for these SNPs were set to missing and would not be analyzed in the statistical tests of association of phenotype and genotypes in a GWA study.

### GWA Statistical Genotype QC

The results of the GWA statistical genotype QC analysis are summarized in Table 3. The results for the 32 specimens are extremely good with a mean per sample genotype missing rate of 0.21%, (99.79% genotype calling rate), and a maximum sample genotype missing rate of 0.64%, (99.36% calling rate). Applying a standard threshold of 95% genotype calling rate for statistical genotype QC analysis would result in none of the 32 samples being dropped after these initial steps. The per SNP missing rates are also comparable with standard results with mean and median missing genotype rates of 0.21% and 0% respectively although the accuracy of the SNP clustering is compromised by availability of only 32 study samples. There were 10,036 SNPs with a missing rate > = 6.25% (> = 2/32 genotypes missing per SNP) and 49,574 SNPs with a missing rate > = 3.125% (> = 1/32 genotypes missing per SNP).

## Discussion

This study was initiated to answer questions about the expected total yield and suitability for GWA analysis of DNA isolated from buffy coat specimens that had been in long term frozen storage for up to 9 years. The buffy coat specimens were collected under the ACCORD trial protocol, from a single participant ACD tube of blood that was drawn, refrigerated, shipped to the ACCORD Central Laboratory on the day of collection, and processed into buffy coats the same day as receipt for long term storage at -80degC. Our concern was the effect of long term storage on total yield and fragmentation of DNA resulting from the denaturation of accompanying blood proteins, or DNA degradation by enzymes released through cell lysis.

We tested two QIAGEN automated protocols for DNA isolation and established a preference for the compromised blood protocol based on apparently lower contamination of the DNA pellets and ease of rehydration, although the yields were similar from this protocol and the buffy coat protocol. The main differences between the protocols are that the compromised blood dispenses greater volumes of reagents for red blood cell lysis,, protein and DNA precipitation and centrifuges for longer during initial DNA pelleting, precipitation and after DNA wash steps. Presumably the increased reagent volumes, particularly the Autopure Precipitation Solution, and the increased centrifugation time account for the lower protein contamination and ease of DNA solution.

We isolated DNA from all 120 buffy coats specimens with variable yields up to a maximum of 312 ug per 8.5 ml ACD tube of whole blood. Only 4 specimens (3.3%) yielded < 10 ug of DNA and 3 of these had 260/280 ratios > 1.9. These 3 samples also had the lowest concentration after the DNA stock dilution procedure (< 21.4 ng/ul) while the 4th had concentration 73.6 ng/ul. The extreme 260/280 ratios may result from low stock concentrations, despite the fact that stated lower limit for the NanoDrop 8000 is 2.5 ng/ul (NanoDrop 8000 Spectrophotometer V2.2 User's Manual, Thermo Scientific Inc), or may reflect the presence of other contaminants that are associated with low yield. Since modern GWA assay protocols require < 1 ug total per sample we expect ample yield from the majority of the samples in storage for GWA studies. Other genetic assays such as next generation exome resequencing using solution-based capture require approx 4-5 ug per sample, but even these more demanding assays should be possible for the majority of samples based on the buffy coat yield distribution.

We found no association of sample race, gender, or regional clinical center network with total DNA yield. We also found no significant association with lab receipt time (range 12-144 hours). We found a dependency on the age of study participant at time of blood draw (-2.1 ug total yield per year of age), which compares with a similar association of approx -5 ug per year of age seen for DNA yields from whole blood samples stored at 4 degC for up to 2.5 years [12]. This decline in yield with age may be caused by a decrease in total leukocyte and lymphocyte count, accompanying a progressive decline in immune function. Erkeller-Yuksel et al found a statistically significant halving in leukocyte count from birth (cord blood) to adults in the 18-year to 70-year age group [13], and studies of reference ranges for lymphocyte subsets have also shown a decline with age [14]. The ACCORD study cohort is comprised of type 2 diabetic patients with comorbidities that could include inflammatory processes, atherosclerosis, and other diabetic complications. Paradoxically, we found that yield significantly increased for the oldest specimens drawn in 2001 after 9 years of storage. It is possible that the 120 test buffy coats may be confounded for year of collection by other clinical factors that could explain the negative dependency of yield on year. The 2001 specimens were from the trial Vanguard phase, and these participants had some clinical differences from those in the main trial phase [8]. In particular they were lighter by 5.6 lbs, and a lower proportion were current smokers (11.4% vs 14.3%). Most intriguing is that there was a big difference in statin use, 45.5% Vanguard vs 61.1% main trial. Pravastatin has recently been shown to decrease the peripheral blood leukocyte count over a six month period in patients with coronary artery disease [15].

For comparison with a recent large study using fresh blood isolation, the international Type 1 Diabetes Genetics Consortium reported that mean total yield of DNA from 14,022 samples of cell pack isolated from 5 ml EDTA tubes of whole blood shipped refrigerated was 144 ug (range 92-165 ug) by salting out or chloroform extraction procedure [16]. The mean yield was 28.8 ug/ml compared to the present study of 15.8 ug/ml, for a 45% loss of yield in comparing the EDTA collected fresh refrigerated blood DNA isolation with that obtained from frozen buffy coat extracted from ACD tubes.

The DNA quality appeared to be very good based on several QC indices including standard agarose gel electrophoresis and TaqMan genotyping of a 6 SNP panel in 117 DNA samples, for a genotyping no-call rate of 1.1% in 702 total genotypes. The missing genotypes were not significantly clustered by SNP or specimen and probably represent instances where desiccation occurred during the PCR reaction for specimens in plate edge wells, hence no unambiguous genotype was registered due to low well sample volume.

The ultimate QC test was whether the samples generated useful GWA genotype profiles for the 32 samples. The resulting DNA produced excellent quality GWA genotyping data as measured by standard post-genotyping statistics. The maximum per sample genotype missing rate was 0.64%. Applying a standard threshold of 95% genotype calling rate for GWA statistical genotype QC analysis would result in none of the 32 samples being dropped after these initial steps. The SNP missing rates are also comparable with standard results with mean and median missing genotype rates of 0.21% and 0% respectively. We substituted 5 of the pre-selected 32 samples for GWA genotyping that had lower total DNA yield (< 50 ug) for higher yield samples and this may have biased the GWA results and the assessment of overall success rate compared to initial random selection, but this step was purely to preserve stock DNA for future disease genetics studies since the DNA in the samples with yield > 10 ug and < 50 ug appeared to have a similar molecular weight profile and integrity compared to the high yield samples. The 4 samples (3.3% of 120 total) with < 10 ug yield had extreme 260/280 ratios (> 2.0) which might suggest possible future problems with genome wide genotyping assays.

## Conclusions

The results of this pilot study demonstrate that buffy coats can be used as a long term clinical trial or biobank specimen for DNA, in lieu of immediately isolating the DNA at collection. We have shown that it can be stored for stored for up to nine years in a -80degC frozen state, yet still produce high yields of DNA that is suitable for GWA analysis and other genetic testing using single SNP genotyping methods.

## Abbreviations

ACCORD: Action to Control Cardiovascular Risk in Diabetes; ACD: Acid Citrate Dextrose; CCN: Clinical Center Network; CNV: Copy Number Variant; dsDNA: double-stranded DNA; GWA: Genome Wide Association; NHLBI: National Heart Lung and Blood Institute; PCR: Polymerase Chain Reaction; QC: Quality Control; SNP: Single Nucleotide Polymorphism

## Competing interests

The authors declare that they have no competing interests.

## Authors' contributions

JCM conceived of the study, participated in its design and coordination and wrote the main draft of the manuscript. EAF performed laboratory genetic assays. JC performed buffy coat laboratory processing and coordinated study specimen repository. JA isolated and performed quality control on DNA. LSL participated in the design of the study and performed data analysis. DWB advised the study on genetic analysis methods and edited the manuscript. SMM designed and coordinated the specimen sample repository and processing protocol. All authors read and approved the final manuscript.
